# An artificial intelligence platform for automated measurement and count estimation of ovarian follicles during ovarian stimulation and IVF: a multicenter study

**DOI:** 10.1007/s10815-025-03777-y

**Published:** 2026-01-03

**Authors:** Piotr Wygocki, Andrzej Zapała, Mateusz Ulfig, Marcin Zieleń, Krystian Zieliński, Natalia Gajewska, Damian Drzyzga, Marcin Wrochna, Piotr Sankowski, Gerard Letterie

**Affiliations:** 1 MIM Fertility, Ul. Świeradowska 47, 02-662 Warsaw, Poland; 2 Institute of Informatics, University of Warsaw, Warsaw, Poland; 3 INVICTA Research and Development Center, Sopot, Poland; 4 Department of Biomedical Engineering, Faculty of Electronics, Telecommunications and Informatics, Gdańsk University of Technology, Gdańsk, Poland; 5 Seattle Reproductive Medicine, Seattle, WA USA

**Keywords:** Artificial intelligence, Reproductive medicine, Computer-assisted image processing, Diagnostic imaging, Ovarian follicle, Automated follicle annotation

## Abstract

**Purpose:**

Ultrasound measurement of follicle diameter is essential in IVF monitoring. This study evaluates the analytical performance of follicle counts and size measurements from two-dimensional images using an AI-based platform, compared to assessments by certified sonographers.

**Methods:**

A total of 5508 TVUS scans from 1689 patients undergoing controlled ovarian stimulation across four IVF centers (Poland, Argentina, Colombia, and the USA) were retrospectively analyzed. All visible follicles were marked using bounding boxes. The dataset included three subsets: training/validation for model development, independent test for evaluating performance across ultrasound systems, and a consensus test set (102 scans from 27 patients) annotated by three expert sonographers. Model performance was assessed using precision, recall, and F1 score. Annotation efficiency was measured by comparing manual and AI-assisted times. Real-world performance was evaluated on a prospective cohort of 904 scans from 269 patients, based on expert adjustments to AI annotations.

**Results:**

For follicles ≥ 10 mm, the model achieved 98.2% precision (95% CI, 96.5–99.2), 88.9% recall (85.0–91.8), and 93.3% F1 score (90.7–95.1). For all follicles, precision and recall were 94.2% (92.8–95.4) and 68.9% (65.9–71.9). Annotation time was reduced 2.5-fold (*p* < 0.01), with an average of 0.54 expert adjustments per scan (CI, 0.47–0.62). Model performance was stable across ultrasound platforms.

**Conclusion:**

This AI platform enables accurate, automated follicle counting and measurement during ovarian stimulation. It matches expert-level performance, improves efficiency, and supports scalable, cost-effective fertility care without compromising quality.

**Supplementary Information:**

The online version contains supplementary material available at 10.1007/s10815-025-03777-y.

## Introduction

Antral follicle count (AFC) is a key predictor of ovarian reserve and response to controlled stimulation during IVF [1–6]. While transvaginal ultrasound (TVUS) is the standard for monitoring, it suffers from high inter-observer variability due to image quality limitations, especially in 2D scans and possible observer fatigue during the monitoring process [7–11]. This subjectivity can lead to measurement errors, affecting care and increasing costs. An AI-based system for automated follicle counting and measurement could reduce variability and costs and enhance accessibility [12].

In conventional ultrasound practice, clinicians do not perform full segmentation (i.e., delineating specific regions of interest). In IVF practice, clinicians place linear diameter measurements on follicles, often focusing on the largest/dominant ones rather than exhaustively measuring every follicle. While this remains the standard of care, the process is time-, labor-, and cost-intensive. Automated analysis may be especially valuable in settings requiring repetitive measurements of small structures, such as antral follicles. Demonstrating feasibility in such contexts could pave the way for broader adoption of automation in clinical imaging. Advanced, AI-enabled software tools hold promises for improving efficiency, reducing cost, and enhancing the sensitivity and specificity of ultrasound interpretation. Automation also eliminates intra- and inter-observer variability. In ovarian stimulation, this approach has strong potential as a stand-alone tool or as part of a broader automated IVF platform. All the above attributes translate to expanded access.

Standard of care for follicle detection and measurement has been classical, hands-on imaging by trained sonographers [13–15]. However, this approach assumes that follicles are uniformly hypoechoic, rounded structures. But images are frequently more complex and complicated by ultrasound artifacts such as acoustic shadowing, atretic follicles, cysts, extra ovarian hypoechoic structures, or non-convex shapes of follicles in proximity to one another. These artifacts can erode the accuracy of interpretation and measurements. In contrast, artificial intelligence, including deep learning models, can mitigate the above shortcomings of conventional scanning and interpretation. It captures context and subtle image changes, which makes it better suited for this task. Prior work applying deep learning to ovarian ultrasound has largely emphasized semantic segmentation, which labels all “follicle” pixels as a single class. Semantic segmentation does not define individual follicle instances; it yields one (or several) contiguous regions without uniquely identifying each follicle. This can be sufficient for coarse quantification of “follicle area”, but it is not adequate for tasks that require instance-level outputs, such as counting distinct follicles and assigning a diameter to each follicle [16, 17]. Many earlier pipelines rely on semantic segmentation followed by heuristic post-processing (e.g., connected components to label contiguous regions and additional separation steps to split touching structures). These steps work when boundaries are clear but can be error-prone when margins are weak or follicles abut—leading to under-segmentation (merging distinct follicles) or over-segmentation. In contrast, our model performs end-to-end instance segmentation, directly outputting per-follicle masks and thereby reducing dependence on such post-processing and the associated merging errors. In this study, we introduce cutting-edge AI solutions to facilitate workflow during IVF and support TVUS for sonographers and fertility specialists to count and delineate all visible follicles during transvaginal ultrasound. The AI model assists the sonographer and clinician in performing examinations during the IVF process by analyzing ovarian scans and generating an automatic report, with robustness across different ultrasound systems. This approach uses 2D cine loops and achieves expert-level performance, validated by comparing model outputs with assessments from three experienced sonographers. The primary objective of the study was to evaluate the analytical performance of an AI system for identification, counting, and measurement of ovarian follicles in routine IVF ultrasound. A secondary objective was to compare the AI with experts across size strata (≥ 10 mm, < 10 mm) and devices/sites. An exploratory objective was to describe factors relevant to clinical integration (workflow compatibility, detection performance, measurement agreement, number of expert edits, device variability).

## Materials and methods

### Dataset

The dataset comprised two components: a retrospective corpus for model development and evaluation, and a prospective corpus for real-time usability and workflow integration. The image library consisted of 5508 TVUS that were collected from 1689 patients at five IVF clinics: one in Poland, two in Argentina, one in Colombia, and one in the USA. Of the 5508 scans, 5341 were 2D cine loops with an average of 74.4 frames per loop and a total of 69,403 annotated follicles. Moreover, 167 scans were 3D data collected from 75 patients, including a total of 1752 annotated follicles. Using a larger 2D cine loop dataset allows the model to achieve high analytical performance for predictions and measurements in 2D cases, while still being capable of handling 3D data. Such dataset structure enabled the network to learn on the most common clinical format while demonstrating extensibility to volumes. All scans were collected during controlled ovarian stimulation cycles as part of IVF treatment under routine clinical conditions without standardized repeat acquisitions or retakes. The scans were acquired using GE Voluson (E6, E8, E10 P6, P8, S8), Samsung HERA W9, and Mindray Z5 ultrasound machines, with resolutions ranging from 649 × 480 to 800 × 1576.

### Study design

To achieve all goals of the study, the patients were divided into three subsets: (I) training and validation dataset (used solely for learning and threshold/hyperparameter selection), (II) test dataset to provide an unbiased estimate of performance and cross-device/site robustness on unseen data, and (III) consensus test dataset. To quantify agreement against a high-fidelity reference, we created (III) a consensus test set: three experts annotated independently and then reached unanimous consensus, enabling size-stratified analyses (AFC on day 1; ≥ 10 mm on days 7–11) and rigorous model-versus-expert comparisons. In parallel, archival counts from routine reports were used to benchmark performance against real-world documentation, acknowledging that late-phase counts may be under-recorded clinically. Finally, in a prospective PACS-integrated cohort, we measured the edit burden (add/modify/delete) during real-time review by sonographers to assess workflow fit. Collectively, this design separates training from evaluation, tests generalization across clinics and machines, and links analytical performance to day-to-day usability. Archival follicle counts were part of the retrospective dataset and served as an independent benchmark against routine clinical documentation, with the caveat that late-stimulation counts may be underestimated.

**Fig. 1:**
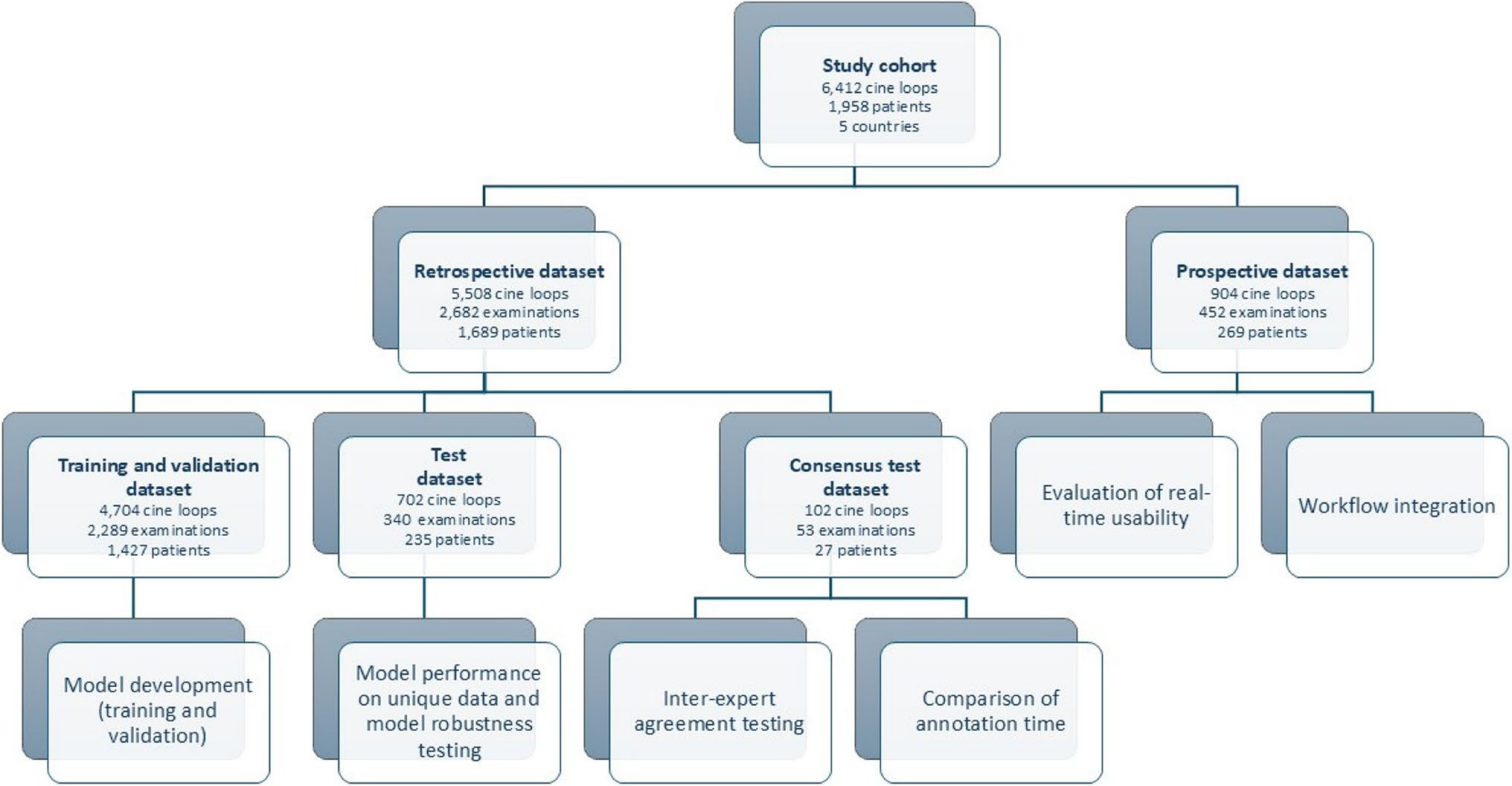
Study design overview. Cohort split into retrospective and prospective datasets, with the former used for model development, independent testing, and expert consensus; and the latter for real-time usability and workflow integration

The study design overview is presented in Fig. 1.

### Retrospective analysis

The characteristics of each subset are detailed in Table 1, while the characteristics of the patients in each group are presented in Table 2.

I. The training and validation dataset comprised a total of 4399 scans from 1372 patients. The remaining set of 305 scans from 55 patients was used as a validation dataset solely to establish prediction thresholds and select hyperparameters for training.
II. The test dataset was used to evaluate the model’s performance on unique data and test the model’s robustness with data acquired from different ultrasound systems. This set included 702 scans collected from 235 patients.
III. The consensus test dataset is a meticulously curated subset designated for in-depth evaluation. The purpose of this dataset was to assess inter-expert agreement. The standard test set was reviewed by one expert per scan, and is therefore subject to that reader’s subjective bias. By contrast, the consensus dataset was created by three experts: all discrepancies were jointly reviewed by the trio, and when disagreement persisted after discussion, a majority (2-of-3) decision was adopted. Twenty-seven patients were selected randomly among all patients with complete data for a single cycle including levels of Anti-Müllerian hormone and estradiol, as well as imaging data from both ovaries on at least two examinations. These included one examination on the first day of ovarian stimulation and another between days 7 and 11, totaling 108 scans. Six of these were excluded because all experts deemed the scan illegible during the annotation process. Two scans were excluded because the ovary was partially cropped, another two because the ovary was entirely not visible, and the final two were rejected due to poor quality, making them unsuitable for accurate follicle measurement. This resulted in 102 scans, with 53 scans from late stimulation and 49 scans from the first day of stimulation. The scans from this dataset were annotated by experts in random order. The comparison of annotation time was performed on the first 25 scans.

**Table 1:** An overview of the retrospective datasets, including the subset names, number of patients, number of scans, number of examinations, and the total count of follicles across each dataset

	Training and validation	Test	Consensus dataset	Total
No. patients	1427	235	27	1689
No. scans	4704	702	102	5508
No. examinations	2289	340	53	2682
No. follicles	57967	11319	1869	71155

**Table 2:** Comprehensive patients’ statistics for each subset, encompassing age, anti-Müllerian hormone (AMH) distribution, the number of IVF cycles, and the outcomes of these cycles, quantified by the number of retrieved MII oocytes

	Training and validation	Test	Consensus dataset
No. patients	1427	235	27
Age (mean ± std)	34.78 ± 5.36	33.49 ± 5.9	33.51 ± 3.88
AMH (mean ± std)	3.11 ± 2.53	4.13 ± 3.68	3.44 ± 2.49
% Low AMH	11.91	7.45	7.41
% Normal AMH	56.21	44.68	51.85
% High AMH	31.88	47.87	40.74
% AMH missing	58.23	60.0	0.0
No. IVF cycles	1581	254	27
No. MII (mean ± std)	7.59 ± 5.49	9.42 ± 6.24	8.59 ± 3.88

Patients are categorized into low (≤ 0.8 pg/ml), normal (between 0.8 and 3.6 pg/ml), and high (≥ 3.6 pg/ml) AMH groups, based on thresholds established in recognized clinical guidelines

### Prospective analysis

The prospective dataset consisted of 904 TVUS cine loops from 452 examinations of 269 patients between November 2023 and February 2024. This dataset was collected as part of a prospective study in which the AI system was integrated into the clinic’s ultrasound system archiving and communication system (PACS). This cohort was designed not to assess clinical outcomes, but to evaluate real-time usability and workflow integration of the AI system under routine conditions. Specifically, it was used to confirm the technical feasibility of PACS integration and instant display, quantify the sonographer’s edit burden (add/modify/delete) as a pragmatic performance measure, and characterize time per scan and common failure modes (e.g., missed small follicles). Cine loops were automatically analyzed by the AI platform, and results were displayed in real time for expert review. In total, 13,710 follicles were evaluated (mean 15.2 follicles per scan). During the review process, the number and nature of edits made by the sonographer to the AI-generated annotations (additions, modifications, or deletions) were recorded to assess performance and to link analytical performance with day-to-day usability.

### Algorithm design

The proposed AI model was designed as a custom model implementing an end-to-end deep-learning approach that directly recognizes specific follicles, eliminating the need for postprocessing and reducing the risk of merging errors characteristic of classical image processing techniques. The model’s input consists of ultrasound scans from TVUS. The scans were recorded before any manual measurements, with sonographers unaware of which images would be used for model development. The model does not require any manual preparation of the input data, such as ovarian location, and operates directly on scans recorded by the ultrasound operator. Detailed information on the model architecture and design, as well as follicles’ detections, is provided in the Supplementary Information (“Model Design” and “Follicle Detection” sections).

### Image annotations

#### Follicle annotations

In the retrospective dataset, each follicle was annotated using bounding boxes delineated in every frame of the video sequence. This was performed by annotators who underwent dedicated training for standardization of the annotation process. All annotations were reviewed and verified by experienced ultrasound specialists to ensure reliability and consistency.

The consensus test dataset was annotated using a separate, more rigorous methodology. In this dataset, each scan was independently assessed by three experts (midwives specializing in ultrasound practice in IVF clinics). Experts A, B, C had respectively 7, 9, and 8 years of professional experience (of which, respectively 5, 6, and 8 years with ultrasound in assisted reproduction clinics). The experts downloaded the DICOM files containing the ovary scans onto their workstations and marked each follicle by manually drawing a reference line. This approach was chosen as it reflects the standard clinical practice for documenting follicle measurements [1, 2]. To establish consensus annotations, the three anonymized, independent assessments were subsequently reviewed in a joint session. During this meeting, the experts systematically examined each follicle and reached unanimous agreement regarding its identification and contours. Using the consensus decision, they defined precise bounding boxes in each relevant frame, ensuring comprehensive detection and enumeration of all follicles present. The steps of the consensus workflow are presented in the Supplementary information Fig. 1.

**Fig. 2:**
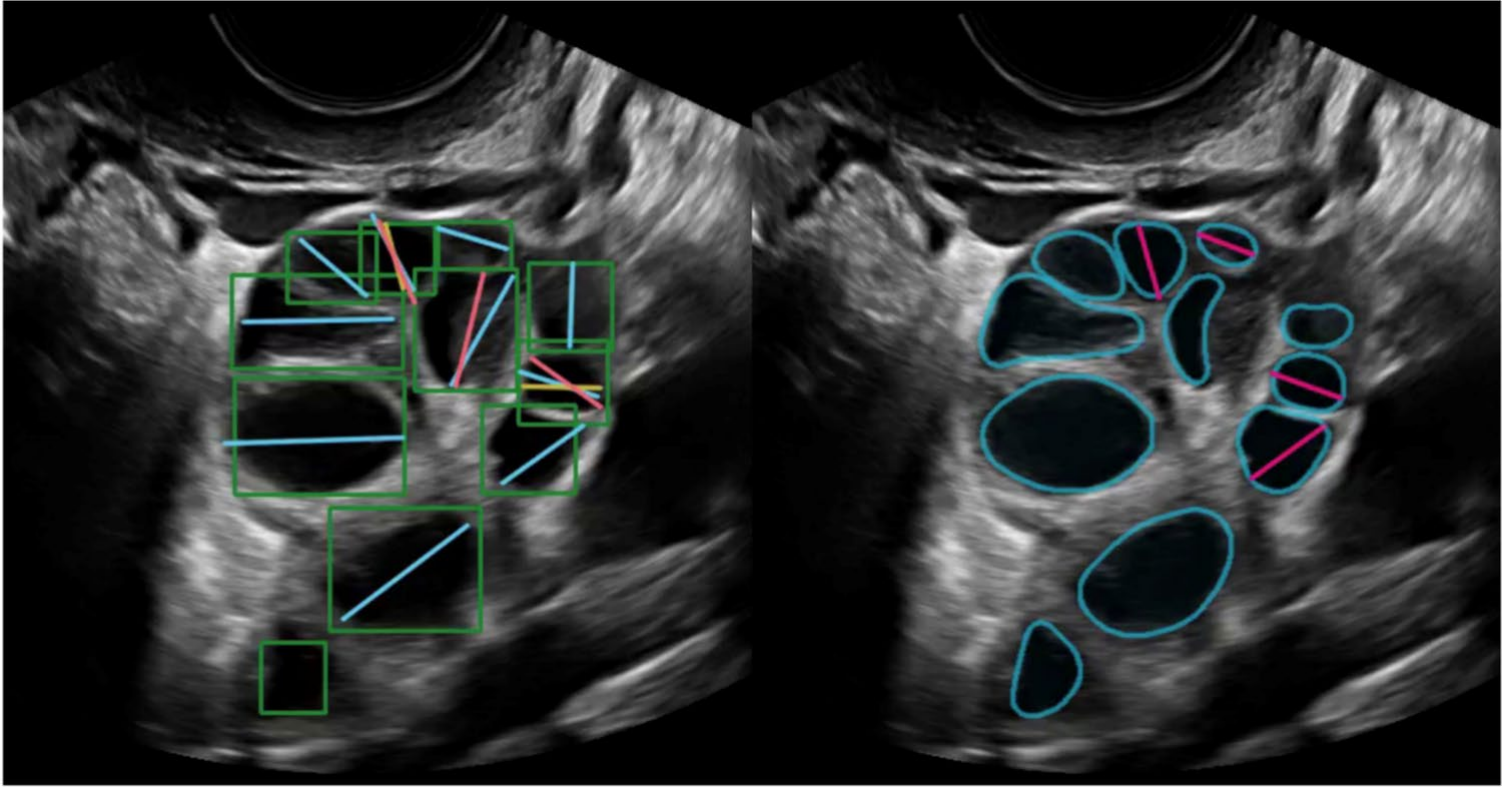
Scan from transvaginal ultrasound, B-mode (brightness mode)/2D Ultrasound. Left: example annotations from three independent experts (blue, yellow, red lines) and their consensus (green boxes). Consensus annotations are bounding boxes around follicles in each frame of the scan. Right, fully automatic outlines and their maximal diameters. Note that line annotations are only made on the frame where the follicle cross-section is judged maximal (experts tend to group these to a few distinct frames)

Importantly, to eliminate potential bias, the AI model’s predictions were not disclosed or utilized at any stage of the consensus process. This procedure created a reference standard enabling objective comparison between expert-derived annotations and automated measurements. Figure 2 presents representative examples of manual expert annotations alongside the corresponding outputs generated by the AI system.

#### Archival follicle counts

Archival follicle counts (retrospective) were retrieved from clinic records made at the time of ultrasound image acquisition, prior to the current study. These records provide an independent comparator free of study-related attention bias. The counts represent the number of follicles identified within specific size intervals, determined by rounding their measured dimensions in millimeters to the nearest even number. These did not include any annotations (follicle locations) and hence could not be used for direct evaluations in terms of precision and recall, but only for agreement comparisons. However, when these counts were made, not all follicles were deemed clinically relevant late in stimulation. Thus, the numbers can be underestimated in the late subgroup and only antral follicle counts on the first day of stimulation can be directly compared.

### Metrics

#### Precision measures and definitions

In order to check whether annotations only include follicles rather than cysts and extra-ovarian structures, we calculated the precision of the model. Precision is the fraction of evaluated annotations that correctly match the reference set; recall (sensitivity) is the fraction of true follicles (in the reference set) that are correctly matched by the evaluated annotations. When evaluating the model in different circumstances, the size threshold can be adjusted below which detections are discarded. Increasing it decreases sensitivity (fewer follicles are found) but could increase precision (as fewer non-follicle structures are found). The precision-recall (P-R) curve was used to demonstrate what trade-offs are available for different thresholds. A balance between precision and recall was achieved by maximizing the F1 score. This score is defined as the harmonic mean of precision and recall, which can also be used to summarize the performance of the model in a single number [18]. Alternatively, the overall performance of a model under different thresholds can be summarized by the area under the P-R curve (often denoted AUC-PR or AP).

#### Follicle counts and sizes

Follicle counts were stratified by size ranges. More specifically, as at a late stage of the stimulation (starting from day 7), the number of large follicles (those that respond to stimulation) is relevant; we used a threshold of 10 mm. Conversely, at stimulation onset, the antral follicle count (AFC) is used to predict response to ovarian stimulation, so follicles (in a single ovary on each scan) of size between 2 and 10 mm were also counted in this study (based on the literature data [1, 19]).

Accordingly, agreement was evaluated between follicle counts generated by the AI model, three clinical experts, and archival clinical reports, using counts derived from consensus annotations as the reference standard within the two subgroups: early and late phases of ovarian stimulation. For completeness and transparency, equivalent comparisons were additionally conducted across the full dataset, irrespective of stimulation timing.

Statistical analysis was made for comparison as follows: Pearson’s correlation coefficient, Lin’s concordance correlation coefficient22 (a coefficient similar to intraclass correlation coefficients, ICC), the mean absolute error (MAE, which is the average of absolute values of differences between follicle counts), and the mean absolute percentage error (MAPE, which is the average of absolute values of ratios between differences and true follicle counts).

In order to validate the precision of follicle diameter measurements generated by the model, we used the consensus dataset, which contains annotations of all visible follicles confirmed by expert consensus. Agreement statistics were subsequently calculated for each pair of experts and for each expert in comparison to the model. Direct comparisons of diameter measurements with the consensus annotations were not conducted, as the consensus data comprised bounding boxes delineating follicle boundaries in each frame, rather than single linear diameter values.

#### Annotation time

To review times of automatic annotations, the time spent creating a text report (folliculogram) on manual annotations (for the first 25 scans from the consensus test dataset) was recorded and compared. One month after finishing all manual annotations, the experts revisited the same 25 scans to review the automatic annotations, ensuring enough time had passed to avoid bias from recalling any details. At this stage, they noted the time spent on the review of results of automatic analysis and corrected any mistakes and omissions. The time taken for generating the automatic annotation by the model was also gathered (processing is done on a server with ordinary 3 GHz CPUs). This study did not record or upload times (for the transfer of scans between the ultrasound and servers or computers), which is usually negligible (roughly 6 s for clinics included in this study). The upload time applied both to automatic and manual measurements in the clinic, as manual measurements in the clinic were also performed on a separate computer.

### Statistical analysis

Two-sided confidence intervals for all metrics were computed via bootstrapping (resampling the scans, with a 95% confidence level, 10′000 resamples, bias corrected). The Wilcoxon Signed-Rank Test was used to prove statistical significance for annotation time speedups. The Python packages used to compute confidence intervals, statistical tests, the area under the precision–recall curve, and correlation coefficients are as follows: scipy (v1.11.1) [20], scikit-learn (v1.3.0) [21], and numpy (v1.24.4) [22].

## Ethics approval

This study was conducted following the approval of the research protocol by the review board of the Regional Medical Chamber in Gdańsk (approval no.: KB – 51/22). Patient information was de-identified before analysis.

### Patient and public involvement

No patients or members of the public were involved in the design, conduct, reporting, or interpretation of this study.

## Results

### Analytical performance

**Fig. 3:**
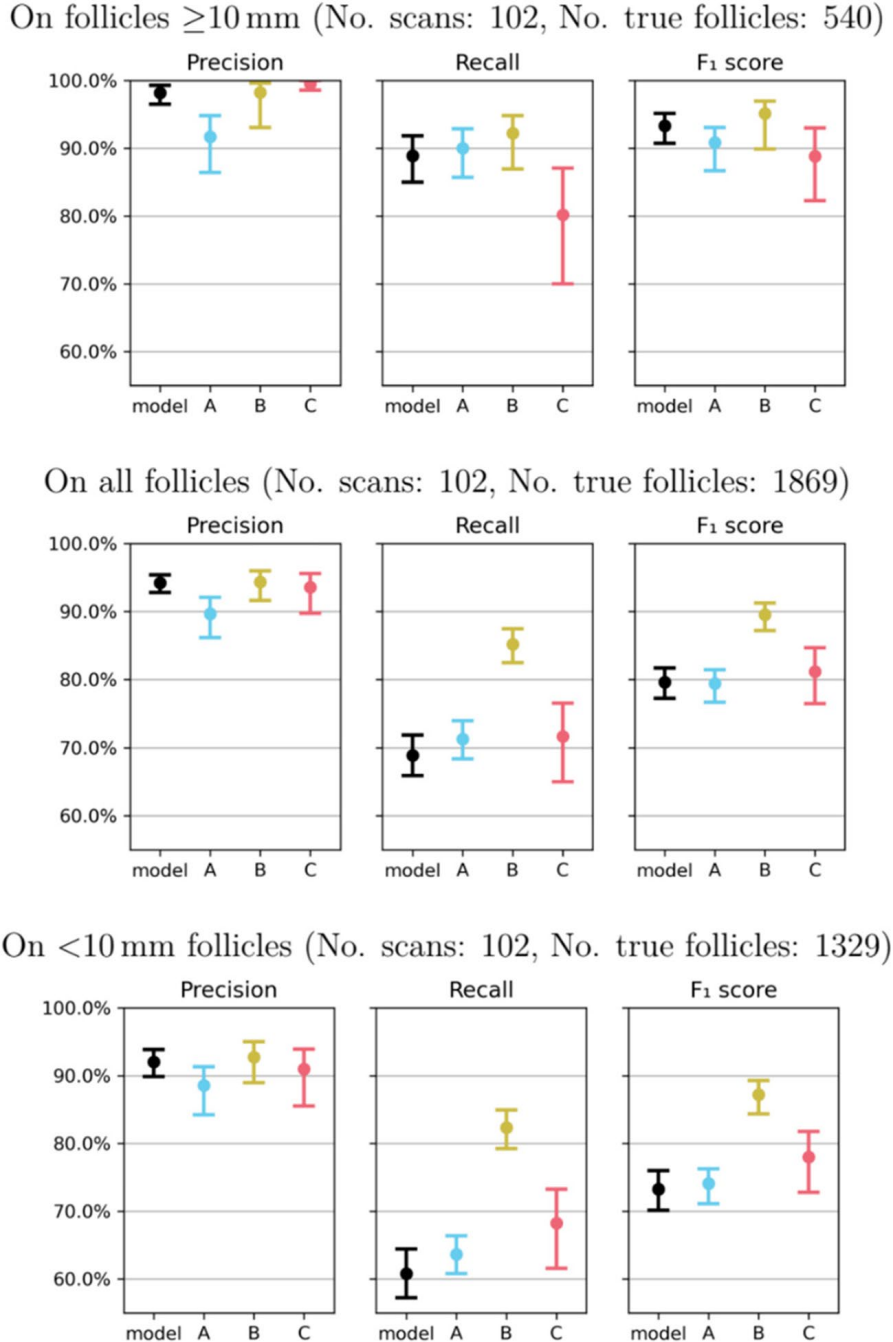
Precision and recall (95% CIs) for the model and Experts A–C on all follicles and by size strata (≥ 10 mm, < 10 mm). The expert average is shown alongside individual experts; the marker indicates the model operating point used for comparisons. (Panels display ≥ 10 mm (top), all follicles (middle), and < 10 mm (bottom)

On the consensus test dataset, considering all follicles, the model showed precision–recall–F1 comparable to the expert average (Experts A–C; Fig. 3, Table 3): performance was similar to two experts, while one expert outperformed the model. Because clinical interpretation differs by phase (AFC at baseline vs stimulation monitoring), we next stratify by size.

**Table 3:** Comparison of precision, recall, and F1 score of AI model and human experts, with 95% confidence intervals (CI)

	Precision (95% CI)	Recall (95% CI)	F1 score (95% CI)
On follicles ≥ 10 mm (no. images: 102, no. true follicles: 540)			
Model	98.2% (96.5–99.2)	88.9% (85.0–91.8)	93.3% (90.7–95.1)
Expert A	91.7% (86.4–94.8)	90.0% (85.7–92.8)	90.8% (86.7–93.1)
Expert B	98.2% (93.0–99.6)	92.2% (86.9–94.8)	95.1% (89.8–97.0)
Expert C	99.5% (98.6–100.0)	80.2% (70.0–87.1)	88.8% (82.2–93.0)
Expert average	96.5%	87.5%	91.6%
On all follicles (no. images: 102, no. true follicles: 1869)			
Model	94.2% (92.8–95.4)	68.9% (65.9–71.9)	79.6% (77.3–81.7)
Expert A	89.7% (86.2–92.1)	71.3% (68.4–74.0)	79.4% (76.7–81.4)
Expert B	94.4% (91.6–96.0)	85.2% (82.5–87.4)	89.5% (87.2–91.2)
Expert C	93.6% (89.8–95.6)	71.7% (65.0–76.5)	81.2% (76.5–84.6)
Expert average	92.6%	76.1%	83.4%
On follicles < 10 mm (no. images: 102, no. true follicles: 1329)			
Model	92.0% (89.8–93.8)	60.8% (57.2–64.4)	73.2% (70.1–76.0)
Expert A	88.6% (84.2–91.3)	63.7% (60.8–66.3)	74.1% (71.1–76.2)
Expert B	92.7% (88.9–95.0)	82.3% (79.2–84.9)	87.2% (84.3–89.3)
Expert C	91.0% (85.5–93.9)	68.2% (61.6–73.3)	78.0% (72.8–81.8)
Expert average	90.8%	71.4%	79.8%

For follicles ≥ 10 mm, the model achieved an F1 of 93.3% (95% CI, 90.7–95.1) with precision 98.2% (95% CI, 96.5–99.2) and recall 88.9% (95% CI, 85.0–91.8), yielding higher precision/recall than in the all-follicles analysis and matching the expert average within overlapping CIs.

For < 10 mm follicles, precision and recall decreased modestly relative to ≥ 10 mm, reflecting the difficulty of small/overlapping structures; here, the model matched two experts, while one expert remained higher (Fig. 3, Table 3).

**Fig. 4:**
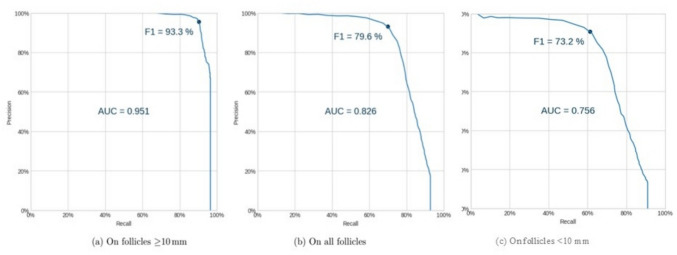
Precision–recall (PR) curve of the AI model across decision thresholds. Area under the PR curve (AUPRC), 0.951 for follicles ≥ 10 mm and 0.826 for all follicles. The dot indicates the F1-maximizing operating point. Precision denotes the fraction of evaluated annotations that correctly match the reference set; recall denotes the fraction of true follicles identified. The reported AUC values summarize overall performance across thresholds

The above results pertain to the model’s default confidence score threshold (obtained by maximizing the F1 score on a validation dataset). Figure 4 shows the precision-recall (P-R) curve of the model for all thresholds. The area under the P-R curve is 0.951 in the case of large follicles. When evaluated on all follicles, regardless of size, the area under the P-R curve is 0.826.

### Agreement in follicle counts (consensus, archival)

**Fig. 5:**
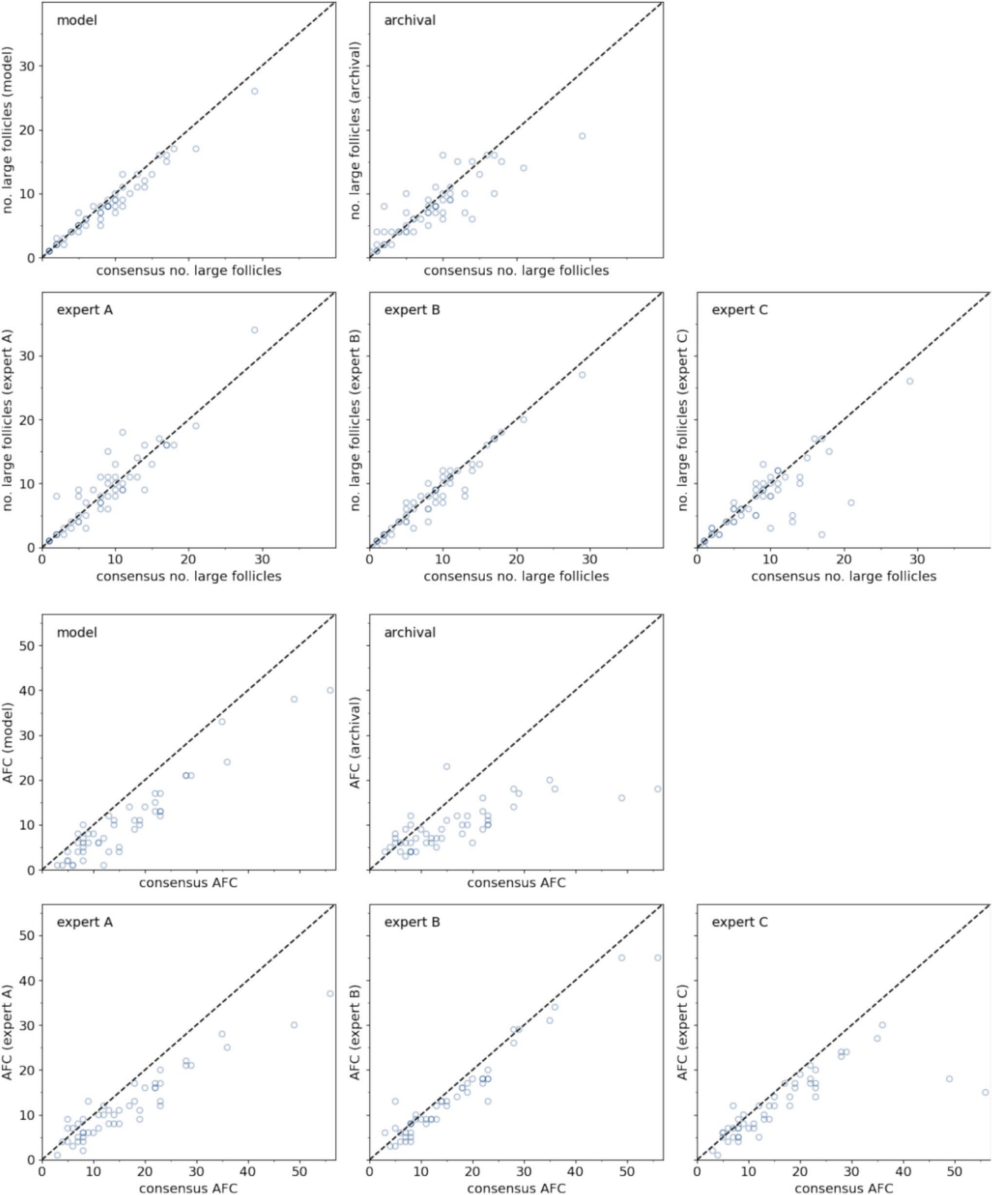
Pair-wise agreement in per-scan follicle counts versus the consensus reference (x-axis). For each panel, the y-axis reports counts from the AI model, archival clinical report, or Experts A–C; the dashed 45° line indicates perfect agreement. Top two rows: counts of large follicles (≥ 10 mm) during late stimulation (days 7–11). Bottom two rows: antral follicle count (AFC) on stimulation day 1. Note that in the < 10 mm panels, points more frequently fall below the identity line, indicating under-count relative to the consensus (consistent with lower recall for small follicles)

To complement detection metrics, we next evaluate agreement in follicle counts per scan against a common reference, because clinical decisions often depend on counts rather than individual detections. For consistency with prior Results, we report two strata: ≥ 10 mm (late stimulation, days 7–11) and < 10 mm (AFC on day 1). Table 4 summarizes count-agreement metrics for ≥ 10 mm and < 10 mm. Figure 5 shows pairwise scatter plots of counts vs the consensus reference (x-axis) for the AI model, archival clinical reports, and Experts A–C (y-axis). For transparency, the same comparisons on all scans (no day constraints) are provided in Table 5. Archival clinical counts are included as an independent, real-world benchmark. For ≥ 10 mm (top two rows of Fig. 5), agreement is higher and dispersion around the identity line is smaller across readers and the model. For < 10 mm (bottom two rows of Fig. 5), points more often lie below the identity line (y < x), reflecting a tendency to under-count small follicles relative to the consensus reference, consistent with our size-stratified detection results. In the AFC (< 10 mm) stratum, all comparators, model and human readers, show modestly lower performance, reflecting the inherent difficulty of detecting small/overlapping follicles; archival clinical reports display the largest negative bias.

**Table 4:** Measures of agreement in follicle counts of annotator (AI model or human expert) with consensus

	Model	Expert A	Expert B	Expert C	archival
Number of large follicles (≥ 10 mm) late in stimulation (days 7–11) (no. scans: 53; no. true follicles: 454)					
Pearson’s corr. coeff	0.98	0.92	0.97	0.80	0.85
Lin’s corr. coeff	0.96	0.92	0.96	0.77	0.81
Mean absolute error	1.02 fol	1.66 fol	1.00 fol	2.02 fol	2.08 fol
Mean abs. percent. error	11.0%	23.3%	14.2%	22.0%	33.4%
Antral follicle count on first day of stimulation (no. scans: 49; no. true follicles: 791)					
Pearson’s corr. coeff	0.95	0.95	0.97	0.78	0.74
Lin’s corr. coeff	0.81	0.80	0.94	0.63	0.41
Mean absolute error	5.67 fol	4.90 fol	2.71 fol	4.43 fol	7.59 fol
Mean abs. percent. error	39.8%	31.5%	22.4%	25.1%	42.2%

**Table 5:** Measures of agreement in follicle counts of annotator (AI model or human expert) with consensus, on all images (regardless of the day of stimulation)

	Model	Expert A	Expert B	Expert C	archival
Number of large follicles (≥ 10 mm) on all images (no. images: 102; no. true follicles: 456)					
Pearson’s corr. coeff	0.99	0.96	0.98	0.90	0.93
Lin’s corr. coeff	0.98	0.96	0.98	0.88	0.92
Mean absolute error	0.53 fol	0.87 fol	0.52 fol	1.05 fol	1.10 fol
Mean abs. percent. error*	10.6%	22.4%	13.6%	21.2%	35.9%
Antral follicle count on all images (no. images: 102; no. true follicles: 1382)					
Pearson’s corr. coeff	0.94	0.93	0.97	0.78	
Lin’s corr. coeff	0.81	0.82	0.94	0.62	
Mean absolute error	4.41 fol	4.02 fol	2.33 fol	4.10 fol	
Mean abs. percent. error	35.1%	31.5%	22.2%	27.2%	

^*^Excludes images with no large follicles

Table 5 extends the pair-wise agreement to all scans (no day constraints), reporting Pearson’s r (linear association), Lin’s CCC (agreement to the *y* = *x* line, penalizing bias and scatter), MAE (absolute count error, in follicles), and MAPE (mean absolute percentage error). Across all scans, r is often similarly high, while MAE can increase because absolute errors grow with larger counts/variance; CCC may fall below r when there is systematic bias (e.g., under-counting < 10 mm; see points below *y* = *x* in Fig. 5). Overall patterns mirror the stratified results: better agreement for ≥ 10 mm, modestly worse for < 10 mm, with larger absolute errors despite similar correlations.

### Measurement agreement (diameter MAE)

Between pairs of experts, the Mean Absolute Error (MAE) for follicles’ sizes measurement was 0.83 mm on average, 95% CI, 0.79 to 0.87 mm (between pairs A–B, A–C, B–C, the MAE was, respectively, 0.97 mm, 0.91 mm, and 0.61 mm). When comparing the algorithm to the experts, the MAE was 0.74 mm on average, 95% CI, 0.70 to 0.78 mm (between the model and respectively A, B, and C, the MAE was 0.93 mm, 0.65 mm, and 0.64 mm), which supports the thesis that the model can constitute a great support for sonography experts.

### Robustness across ultrasound machines

We compared performance across five ultrasound systems with differing native resolutions (E8, 960 × 720; S8, 975 × 743; E10, 1136 × 852; HERA W9, 1276 × 800, 1576 × 800, 640 × 480; Z5, 800 × 600). On the independent test set (all follicles), performance was stable with overlapping 95% CIs across devices: overall precision 92% (91–93%), recall 86% (85–87%), F1 89% (88–90%), *N* = 702. By device: E8 92/85/88 (*n* = 358), S8 92/84/88 (*n* = 21), E10 92/84/88 (*n* = 20), HERA W9 94/88/91 (*n* = 202), Z5 94/82/88 (*n* = 101). Differences were not clinically meaningful given CI overlap. Note that these test-set summaries (all follicles, micro-averaged across scans) are not directly comparable to Fig. 3, which reports the consensus subset with exhaustive small-follicle annotations and model–expert operating-point comparisons; the distinct datasets/averaging explain higher “all-follicles” values here. The results presented in Table 6 indicate the model’s stable performance across all apparatus.

**Table 6:** Comparison of precision, recall, and F1 score of AI model on scans from different ultrasound machines (on all follicles, regardless of size)

Machine	*N*	Precision (95% CI)	Recall (95% CI)	F1 (95% CI)
Voluson E8	358	92% (90–92%)	85% (84–87%)	88% (87–89%)
Voluson S8	21	92% (89–95%)	84% (79–87%)	88% (84–91%)
Voluson E10	20	92% (83–98%)	84% (77–92%)	88% (81–94%)
Samsung HERA W9	202	94% (92–95%)	88% (87–90%)	91% (90–92%)
Mindray Z5	101	94% (92–96%)	82% (78–86%)	88% (85–90%)
Overall	702	92% (91–93%)	86% (85–87%)	89% (88–90%)
Machine	Resolutions			
Voluson E8		960 × 720		
Voluson S8		975 × 743		
Voluson E10		1136 × 852		
Samsung HERA W9		1276 × 800, 1576 × 800, 640 × 480		
Mindray Z5		800 × 600		

### Annotation time (manual vs AI-assisted)

**Fig. 6:**
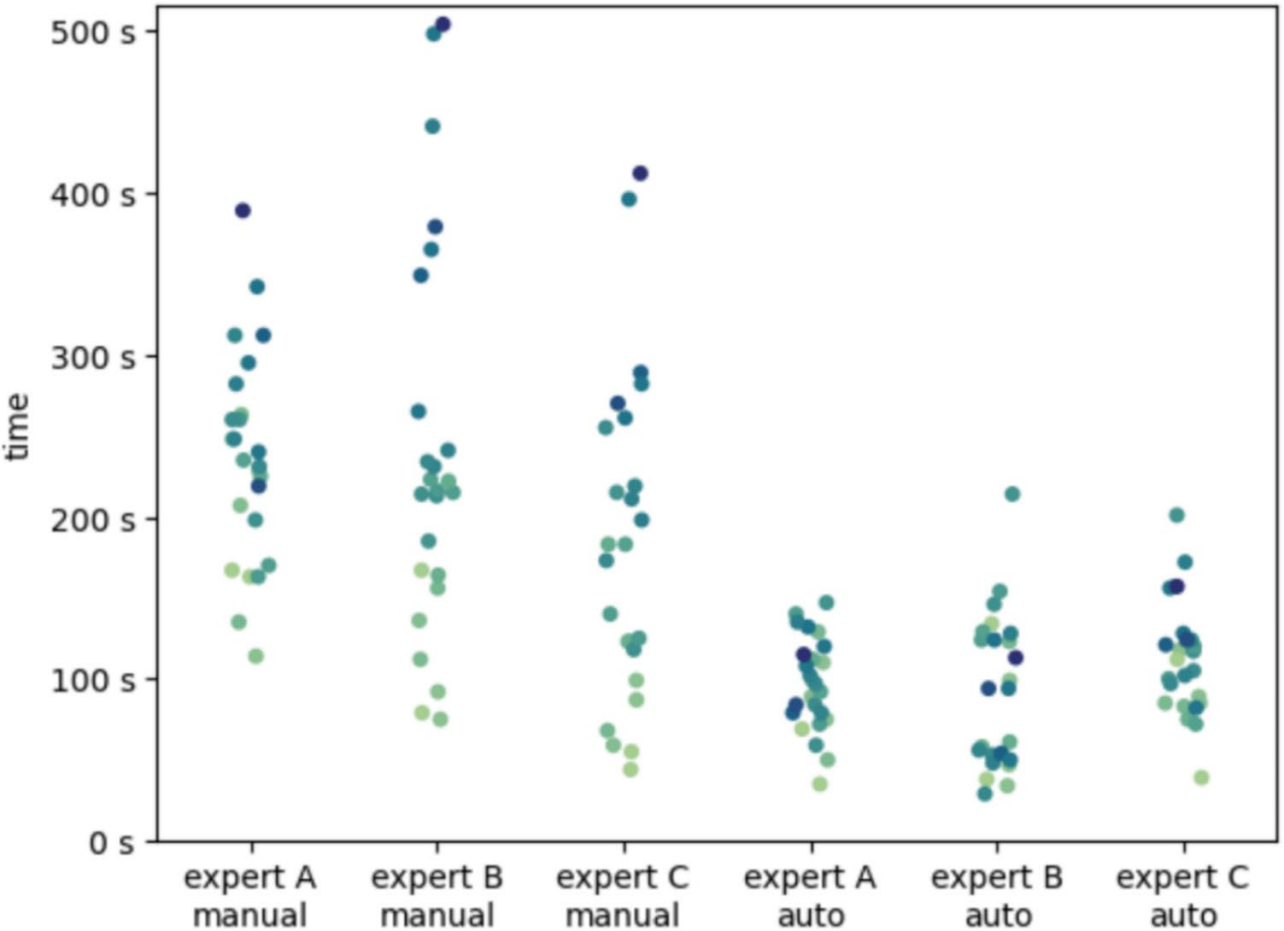
Comparison of time spent on manual follicle annotation vs reviewing automatic annotations (these include the time of the automatic processing and the time of correcting any missing or faulty annotations). Darker colors indicate videos with more follicles

The average time spent on manual annotations (per ovary) as measured on the first 25 scans of the consensus dataset was 220 s (3 min 40 s), similar to what is reported in the literature [10, 31, 35]. Using the Wilcoxon Signed-Rank Test, the average speed-up (defined as manual time/review) across scans was found to be at least 2.5 ×, and this difference was statistically significant (*p* < 0.01). The automatic processing itself required on average 20 s (performed on standard 3 GHz CPUs). When the review time was considered, the overall time savings remained substantial, with an average speed-up factor (defined as manual time/automatic processing + review) of 2.2 × and a minimum observed speed-up of 2.0 ×, statistically significant (*p* < 0.024). The speed-up factor for individual experts varied from 1.9 × to 3.5 ×. There was an even larger variability in individual scans. Table 7 summarizes the statistics for each expert. Figure 6 shows the time spent on manual and assisted annotations (automating processing plus review time) for all 25 cases. Manual annotations were more time-consuming, often several-fold for videos with more follicles, than annotations obtained with the help of AI, which included time needed for automatic processing and for correcting any missing or faulty annotations. Time savings were most pronounced in scans with a higher follicle burden; residual edits were more frequent for < 10 mm follicles, reflecting the greater difficulty of small/overlapping structures and aligning with the size-stratified detection results.

**Table 7:** Comparison of manual annotation times, automated processing times, and review times for 25 scans from the consensus test dataset

	Expert A	Expert B	Expert C	Overall
Avg time manual annotation [s]	239.0	184.0	236.0	220.0
Avg time review with processing AI annotations [s]	91.0	113.0	97.0	100.0
Avg time automatic processing annotations [s]	23.0	18.0	19.0	20.0
Avg time reviewing automatic annotations [s]	68.0	95.0	78.0	80.0
Avg time gained [s]	171.0	88.0	159.0	139.0
Speedup (manual/review)*	3.5	1.9	3.0	2.7
Speedup (manual/review with processing)*	2.6	1.6	2.4	2.2

The table highlights the time saved by using the AI model, the speed-up factors achieved across individual experts, and the mean values for all experts

^*^Speed-up value is manual time/AI-assisted total time where AI-assisted total time = automatic processing time + expert review time. Values > 1 indicate time savings with AI (faster than manual); values = 1 indicate parity, and values < 1 indicate slower than manual

### Prospective evaluation (real-time usability and workflow integration)

To explore clinical integration and the average number of edits by sonographers during automated measurements, we quantified edit burden after real-time AI analysis in routine practice. Among all 904 scans collected prospectively, where AI annotations were later reviewed by sonography experts performing the examination, the average number of editions (follicle additions, modifications of measurement, or deletions) was 0.54 per scan (95% CI, 0.47–0.62). At least one edit was made in 29% of scans (95% CI 26–32%; 265/904). In total, 3.57% of all follicles were edited in any way (CI, 3.27–3.90%; 490/13710). The 490 follicle editions can be characterized as follows: 1.52% follicles were added (CI, 1.33–1.74%; 209/13710), 0.66% were modified (CI, 0.53–0.81%; 91/19710), and 1.39% were deleted (CI, 1.20–1.60%; 190/13710). By diameter on the index frame, edited follicles were distributed as: 2–5 mm (18%), 5–10 mm (28%), 10–15 mm (29%), 15–20 mm (20%), and ≥ 20 mm (4%). The low overall edit rate supports workflow fit and precision of the AI output for most follicles, while the enrichment of edits in smaller diameters is consistent with the known difficulty of < 10 mm structures on 2D TVUS and highlights where targeted improvements or brief review remain valuable.

## Discussion

The algorithm enables automated counting and measurements of follicle diameters in two dimensions. In brief, the system first locates regions likely to contain follicles, then outlines each follicle and follows it across the cine loop. This two-step approach mirrors clinical reading: detect the structure, then delineate it carefully. Measurements are taken on the frame where a follicle is largest, which is normally performed in clinical practice.

First, the process of routine follicle measurements could be automated with little or no need for the painstaking process of manual caliper placement and measurements. Automatic methods can address key limitations of manual annotations, which are inherently constrained by intra- and inter-observer variability, differences in operator experience, and subjective interpretation [23]. High-quality manual annotations are also challenged by limited image resolution, acoustic artifacts, and frequent omission of small follicles, particularly in complex cases such as polycystic ovary syndrome [24]. These inconsistencies can undermine reproducibility and impact clinical decisions where precise follicle counts are essential. Integrating AI-assisted annotation systems offers the potential to standardize measurements, reduce variability, and improve efficiency. Importantly, automatic annotations create a consistent, traceable record of each scan and measurement. This objective documentation supports transparency, facilitates treatment audits, and provides clear justification for clinical decisions. In cases where retrospective review is necessary, for example, to reassess a patient’s response or address medicolegal questions, such records can be invaluable.

This design helps the algorithm focus on clinically relevant objects, reduce false positives, and produce consistent size estimates across different machines and operators.

On follicles measuring at least 10 mm in diameter, the fully automated measurement and counting achieved by the algorithm AI model demonstrated performance comparable to that of human experts, supporting its potential for self-sufficient follicle monitoring during stimulation. For smaller follicles, model performance was similar to some experts, though slightly inferior to the most experienced one. This finding was corroborated in the prospective trial, where only a small number of edits were required.

Automated detection of large follicles closely matched expert consensus. For smaller (2–10 mm) follicles, automated antral follicle counts were consistent and correlated well with reference values, though with slightly higher error margins than experts. The differences were minor, suggesting automated AFC may be clinically acceptable. Comparisons with archival data further suggested that routine expert assessments may occasionally omit some follicles.

Taken together, these results indicate performance comparable to individual experts overall, with the greatest concordance for ≥ 10 mm follicles and expected dispersion for small follicles.

The retrospective analysis was limited by the labor-intensive consensus annotation process, but it could be replicated in other clinics with varying ultrasound and follicle measurement protocols (e.g., use of two perpendicular lines). Although labor-intensive, using three expert annotations and a consensus process yielded a more robust reference standard. Unlike prior studies based on single-expert input, this approach accounts for inter-observer variability and reduces systematic bias from missed or ambiguous follicles.

An ancillary observation enabled by consensus annotations was the frequent omission of small follicles (< 10 mm) by experts. This suggests that the challenges of difficult scans may have been underestimated and underscores the need for improved enumeration methods. While prior studies reported moderate to good inter-observer agreement in manual counts [11, 23], variability increases with higher follicle numbers, and some have shown poor concordance (e.g., Lin’s coefficients ranging from 0.08 to 0.63) [8], particularly in polycystic ovaries [9, 10]. Furthermore, agreement metrics (Pearson’s, Lin’s, and intraclass correlations) are influenced by dataset variance and outliers, making comparisons across studies difficult.

Second, it has important workflow implications, with scan time being significantly reduced and precision improved. This time reduction has important implications for savings and streamlining the tedious daily process of assessments of the IVF process. This should not be interpreted as reducing the importance of personalized care and direct patient–provider communication. It does suggest that, through an automated process, the time may be better spent in face-to-face discussion if needed or desired by the team or patient.

Further study showed that with most follicles automatically annotated, experts were able to complete the procedure substantially faster, often several times quicker. Excluding the time required for automatic processing, which allows the sonographer to attend to other tasks, the efficiency gains would be even greater.

In practice, very experienced sonographers still performed targeted edits in a minority of cases (small, irregular, or borderline-quality images), indicating where further refinement is most useful.

The only comparable published solution for automated follicle detection is SonoAVC (GE), considered semi-automatic [2] due to required manual preprocessing (ovary selection) and postprocessing (review). Its performance is strongly dependent on image quality [25, 26]. In one study, mean antral follicle counts obtained automatically were three times lower than manual counts [27]. A more recent evaluation reported a recall of 53% and a mean absolute error of 1.4 mm in follicle diameter on 3D scans [28]. Nonetheless, SonoAVC has been shown to improve inter-observer reliability and annotation times when high-quality 3D images are available [26, 29]. A key limitation of this study is that consensus annotations remain inherently biased toward human perception and do not represent perfect ground truth. Consensus was established without knowledge of AI outputs and may reflect what is easier for humans to identify, particularly given the similar training background of the annotators. Although experts were instructed to mark all visible follicles regardless of perceived clinical relevance, further divergence could occur in routine practice. Additionally, while experts routinely analyzed images offline, the data collection for this study, requiring multiple independent annotations, may have introduced attention bias that increased their performance but extended annotation time. This is reflected in comparisons between expert and archival AFC. These design choices were intentionally conservative to enable a rigorous and critical evaluation of the model, accepting bias against the AI approach when unavoidable.

In summary, the study describes the first iteration of software for automated follicular measurement during ovarian stimulation and proposes that evolving algorithms represent a valuable tool in clinical practice. This version of the software was effective in analyzing large, symmetrical follicles with high-quality imaging but demonstrated reduced performance for smaller follicles, those with irregular borders, or in out-of-focus images. These limitations will be addressed in the second iteration through software enhancements, including shape-based constraints for boundary detection and optimized ultrasound image acquisition. Computerized image measurements have the potential to reduce time and inter- and intra-observer variability and improve workflow and patient care, demonstrating expert-level performance across diverse clinical datasets and ultrasound platforms. The development of a validated annotation framework is essential for studies evaluating AI methods.

## Supplementary Information

Below is the link to the electronic supplementary material.

ESM 1 DOCX (2.82 MB)

## Data Availability

The ultrasound images collected in this study are not publicly available due to patient privacy and data-protection regulations. A de-identified dataset (100 TVUS cine loops) and time-limited access to the current, versioned inference API are available from the corresponding author upon reasonable request for non-commercial research under a data-use agreement. As the production model is actively maintained and improved, API outputs may match or exceed the performance reported here. The AI system is commercially available as part of FOLLISCAN; training code and the full training dataset are not shareable.
