# Supplementary material for: An artificial intelligence platform for automated measurement and count estimation of ovarian follicles during ovarian stimulation and IVF: a multicenter study
Source: J Assist Reprod Genet. 2026 Jan 3;43(3):715–29. doi: 10.1007/s10815-025-03777-y (PMC12982739; doi:10.1007/s10815-025-03777-y)
Supplement: Supplementary file 1 — DOCX (2.82 MB) [file 10815_2025_3777_MOESM1_ESM.docx]

**Supplementary information**


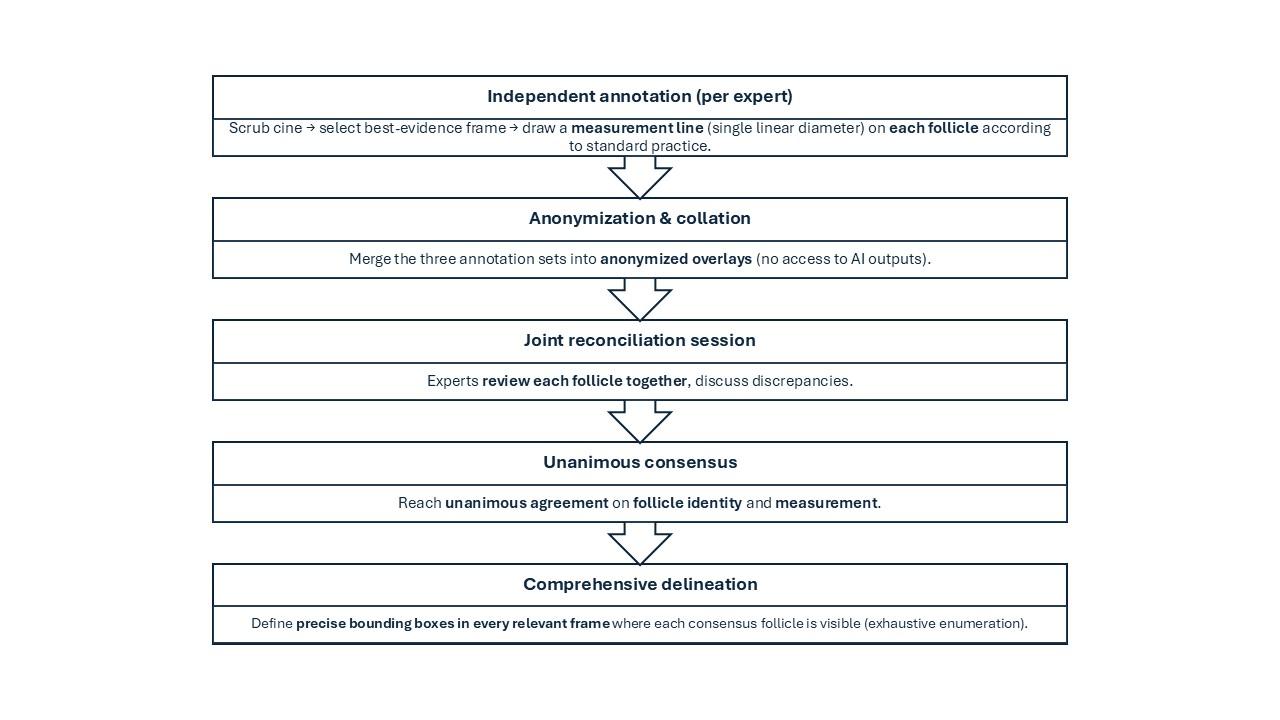


Supplementary information Figure 1. Consensus annotation workflow performed by three experts.

1. **Model Design**

The model architecture employs a U-Net backbone [1] (containing convolutional layers and residual connections), widely used in medical computer vision. This backbone is coupled with modules for classification, which estimate the likelihood of a region containing a follicle and indicate the bounding box of that region. These modules are based on the YOLO architecture and its later improvements [2-4]. To allow the model to identify exact 3D outlines of each follicle individually we extended these with an instance segmentation module inspired by the Mask R-CNN architecture19. This module extracts specific portions of the feature maps corresponding to the predicted bounding boxes and generates segmentation masks using convolutional layers. The model produces complete follicle outlines across all frames where each follicle appears, enabling the identification of individual follicles. For each follicle, the size measurement is determined by identifying the longest diameter on the frame showing the largest follicle cross-section, consistent with standard clinical practice. Training used stochastic gradient descent optimization with Nesterov momentum [5]. Training the model with 85M parameters required 66 hours on 4 GPUs.

1. **Follicles Detection**

In order to evaluate manual and automatic annotations in the same way, the comparison was made against consensus annotations. As mentioned before consensus annotations served as the ground truth. The methodology outlined below follows the standard practices commonly used in object detection tasks [6,7]. An evaluated annotation (manual or automatic) is considered to be correct if it matches a ground truth annotation with sufficiently close and similar bounding boxes. Specifically, we require the Intersection over-Union (IoU, or Jaccard index) to be at least 35 %. In previous work, IoU thresholds used for cine loops or 3D data were set to 35 %46, 30 %48 or even 0 % (i.e., any intersection is sufficient for a match)30. Since evaluated annotations (manual or automatic) are given in the form of lines, they are first circumscribed in a circle to obtain a bounding box on a single frame. The ground truth annotations consist of bounding boxes defined for each frame, and the IoU is computed using the bounding box corresponding to the frame of the evaluated annotation. Optimal (Hungarian) matching between the boxes was used [6,7]. In this way, the evaluated annotation can only match to one consensus annotation, so that duplicate or split findings are counted as errors. Similarly, only one evaluated annotation can match each consensus annotation, so that fused findings are counted as errors.

**Supplementary material references**

1. Ronneberger O, Fischer P, Brox T. U-Net: Convolutional networks for biomedical image segmentation. In: Navab N, Hornegger J, Wells WM, Frangi AF, editors. Medical Image Computing and Computer-Assisted Intervention – MICCAI 2015. Lecture Notes in Computer Science, vol. 9351. Cham: Springer; 2015. p. 234–41. https://doi.org/10.1007/978-3-319-24574-4_28
2. Redmon J, Divvala S, Girshick R, Farhadi A. You only look once: Unified, real-time object detection. In: Proceedings of the IEEE Conference on Computer Vision and Pattern Recognition (CVPR); 2016. p. 779–88. https://doi.org/10.48550/arXiv.1506.02640
3. Redmon J, Farhadi A. YOLO9000: Better, faster, stronger. In: Proceedings of the IEEE Conference on Computer Vision and Pattern Recognition (CVPR); 2017. https://doi.org/10.48550/arXiv.1612.08242
4. Jiang P, Ergu D, Liu F, Cai Y, Ma B. A review of YOLO algorithm developments. Procedia Comput Sci. 2022;199:1066–73. https://doi.org/10.1016/j.procs.2022.01.135
5. Sutskever I, Martens J, Dahl G, Hinton G. On the importance of initialization and momentum in deep learning. In: Proceedings of the 30th International Conference on Machine Learning (ICML); 2013. Vol. 28(3). p. 1139–47. https://proceedings.mlr.press/v28/sutskever13.html
6. Reinke A, Eisenmann M, Tizabi MD, et al. Common limitations of performance metrics in biomedical image analysis. Med Image Deep Learn. 2021. https://doi.org/10.48550/arXiv.2104.05642
7. Maier-Hein L, Reinke A, Godau P, et al. Metrics reloaded: Recommendations for image analysis validation. Nat Methods. 2023;21:195–212. https://doi.org/10.1038/s41592-023-02151-z
